# Associations between depression, fear of COVID-19 infection and students’ self-care measures used during the first wave of the pandemic

**DOI:** 10.1186/s12889-023-15954-8

**Published:** 2023-06-01

**Authors:** Passent Ellakany, Morenike Oluwatoyin Folayan, Maha El Tantawi, Roberto Ariel Abeldaño Zuñiga, Nourhan M. Aly, Eshrat Ara, Balgis Gaffar, Anthonia Omotola Ishabiyi, Mir Faeq Ali Quadri, Abeedah Tu-Allah Khan, Zumama Khalid, Folake Barakat Lawal, Bamidele Olubukola Popoola, Joanne Lusher, Muhammad Abrar Yousaf, Jorma I. Virtanen, Annie Lu Nguyen, Mental Health and Wellness study group

**Affiliations:** 1 Department of Substitutive Dental Sciences, College of Dentistry, Imam Abdulrahman Bin Faisal University, Dammam, Saudi Arabia; 2 Department of Child Dental Health, Obafemi Awolowo University, Ile-Ife, Nigeria; 3 Department of Pediatric Dentistry and Dental Public Health, Faculty of Dentistry, Alexandria University, Alexandria, Egypt; 4 Postgraduate Department, University of Sierra Sur, Oaxaca, Mexico; 5 Department of Psychology, Government College for Women, Cluster University of Srinagar, Moulana Azad Road Srinagar Kashmir, Jammu and Kashmir, 190001 India; 6 Department of Preventive Dental Sciences, College of Dentistry, Imam Abdulrahman Bin Faisal University, Dammam, Saudi Arabia; 7 Department of Sociology, University of Cincinnati, Cincinnati, Ohio USA; 8 Department of Oral Health Sciences, School of Dentistry, University of Washington, Seattle, WA USA; 9 School of Biological Sciences, University of the Punjab, New Campus, Lahore, 54590 Pakistan; 10 Department of Periodontology and Community Dentistry, College of Medicine, University of Ibdan, Ibadan, Nigeria; 11 Department of Child Oral Health, University of Ibadan, Ibadan, Nigeria; 12 Regent’s University London, London, UK; 13 Department of Biology, Virtual University of Pakistan, Lahore, Pakistan; 14 Faculty of Medicine, University of Turku, Turku, Finland; 15 Department of Family Medicine, Keck School of Medicine, University of Southern California, Los Angeles, CA USA

**Keywords:** COVID-19, Students, Self-care, Depression, Fear of COVID-19, Coping

## Abstract

**Background:**

COVID-19 lockdown resulted in the closure of schools with associated problems. The aim of this study was to determine the associations between depression, fear of contracting COVID-19 infection and the use of self-care measures by college students during the first wave of the COVID-19 pandemic.

**Methods:**

This was a cross-sectional study that collected data from undergraduate and postgraduate college students 18 years and older from 152 countries between June and December 2020. Study participants were recruited through crowdsourcing using various social media platforms including Facebook, Twitter, and Instagram, WhatsApp groups and emails to participants in the collaborators’ networks. The dependent variables were fear of contracting COVID-19 and depression while the independent variable was students’ self-care measures. Multivariable logistic regression models were conducted to assess the associations between the dependent and independent variables.

**Results:**

Of the 2840 respondents, 1305 (46.0%) had fears of contracting COVID-19 and 599 (21.1%) reported depression. The most common self-care measures were phone calls with friends/family (60.1%) and video chat (52.8%). Learning a new skill was significantly associated with higher odds of fear of contracting COVID-19 (AOR = 1.669) and lower odds of having depression (AOR = 0.684). Talking to friends/family through video chat (AOR = 0.809) was significantly associated with lower odds of feeling depressed while spending time with pets (AOR = 1.470) and taking breaks from the news/social media (AOR = 1.242) were significantly associated with higher odds of feeling depressed. Students from lower middle-income countries (AOR = 0.330) had significantly lower odds of feeling depressed than students from low-income countries.

**Conclusion:**

Self-care strategies involving social interactions were associated with less depression. Coping strategies with more cognitive demands may significantly reduce the risk of fear of COVID-19. Special attention needs to be given to students in low-income countries who have higher odds of depression during the pandemic than students from other countries.

## Background

More than 90 countries used partial or complete lockdown to control the COVID-19 pandemic during the first wave [1]. The pandemic and the lockdown measures have had several negative impacts on the global economy, educational system and health care delivery [2, 3]. Many people have experienced mental stress resulting from fear and concern about contracting and transmitting the infection [4, 5]. Others have suffered mental health challenges due to limited access to support systems, and the need for behavioural modification to adapt to COVID-19 preventive measures [6].

Lockdown resulted in the closure of many higher education institutions [7–9]; and many of these resorted to virtual online education to protect students from viral transmission [8]. Students were highly affected during the lockdowns and many of them faced mental health distress due to being isolated as a result of staying at home, and suffering loneliness and feeling disconnected whilst staying away from roommates, peers and staff [10, 11]. Moreover, students faced feelings of stress, anxiety and depression due to COVID-related restrictive measures [6, 9, 10], in addition to concerns about delays with graduation and job opportunities [12, 13]. The loss of part-time work and parental income as a result of the pandemic also caused financial problems for some students as they became unable to raise funds for education fees during lockdown [14]. These financial problems might have worsened the mental health status of many students [15].

Mental health problems associated with COVID-lockdown may have been further aggravated by poor access to mental health counselling services that were available during pre-pandemic academic sessions, poor access to online counselling centres during lockdown and unwillingness to build a relationship with a new or unfamiliar counselling service [12, 14]. The fear of contracting COVID-19 infection [4] might also have magnified negative feelings and worsened the mental health impact of the pandemic [16]. There is a complex relationship between fear of contracting COVID-19, stress, and anxiety, and the development of depression symptoms in students [17]. Depression might be directly and positively associated with fears and stress relating to contracting COVID-19; and might also be indirectly associated with stress and fears of contracting COVID-19, mediated by anxiety [17]. Depressive symptoms among students were also found to be associated with social distancing and lockdown [11, 18, 19].

Although students are faced with increased risk of mental health problems during the pandemic, it is likely that they may have adopted self-care coping measures. This may include new ways of socialising with families and friends to address boredom, distress and irritability [18]. Active coping (self-care) measures include establishing virtual communication through phone or video calls, watching movies, concerts and museums, exercising at home, performing online yoga, meditation, cooking, knitting and reading books [18, 20].

Several factors could potentially affect the adoption of self-care measures. These include perception of the added value of the solution [21] in addition to morality [22], social factors such as educational status, housing, financial status, and family environment in addition to cultural factors such as discrimination against gender in certain cultures [23–25]. The effect and magnitude of these factors may differ by country income level as indicated by the differences in avoidance behaviors between nursing students in Australia and India [9]. There is, however, little known about how the fear of contracting COVID-19 or depression during the COVID-19 pandemic may influence the choice of self-care strategies; or how context may affect the choice of these self-care measures among students.

The aim of the present study was to determine the associations between depression, fear of contracting COVID-19 infection and the use of self-care measures by college students during the first wave of the COVID-19 pandemic. We hypothesized that students with depression and fear of contracting COVID-19 would be more likely to adopt self-care measures than those who do not have depression and fear of contracting COVID-19. The study also hypothesized that country income level would be associated with having depressive symptoms and fears of COVID-19.

## Methods

### Ethics approval

Ethical approval for the study was obtained from the Human Research Ethics Committee at the Institute of Public Health of the Obafemi Awolowo University Ile-Ife, Nigeria (IPHOAU/12/1557). Additional ethical approvals were obtained from India (D-1791-uz and D-1790-uz), Saudi Arabia (CODJU-2006 F), Brazil (CAAE N° 38423820.2.0000.0010), Bioethics Committee, University of the Punjab, Lahore, Pakistan (158,042) and the UK (13,283/10,570) [26].

### Study design and population

This was a secondary analysis of data collected from 152 countries on mental health and wellness using an online survey. The survey data was collected between 29th June 2020 until the 31st of December 2020. The study recruited respondents 18 years and above who were able to read, had access to the internet and consented to participate in the study. Details on the study methods and profile of the participants have been published earlier [26].

The population for the present study were restricted to respondents who self-reported as “undergraduate” and “postgraduate” college students as their current status. There were 2,840 respondents with complete data extracted from the database of the primary study.

### Sample size

The pre-survey minimum sample size for this study was based on the estimated prevalence of the most common global mental health disorder in 2019 (3.94% for anxiety disorder) [27]; a desired precision of estimate = 0.05 and a confidence level = 95% for an infinite population size [28]. The sample size was increased by 10% to allow for missing responses [29]. Based on statistical modelling, sample size was adequate when there was a minimum of 10 participants with complete responses per each of the independent variables for the study to allow regression analyses with a minimum probability level (p-value) of 0.05 [30].

### Recruitment procedure

Data was collected using an online survey platform, SurveyMonkey®. Web-based data collection was an appropriate modality for collecting data during the COVID-19 pandemic when movement was restricted as a public health intervention [31]. Each participant could only answer the questionnaire once. Participants had the ability to edit their responses freely until they decided to submit. Email addresses were not collected to gurantee the anonymity of the responses. Participants were recruited using various social media platforms (Facebook, Twitter, and Instagram), WhatsApp groups and emails to participants in the networks of 49 collaborators.

### Instrument validation

The instrument used for the survey was assessed for content validity in addition to other evaluations as described in detail in previous studies [26, 32]. The overall content validity index of the survey was 0.83. Responses collected for content validation were excluded from the final analysis [32].

### Data collection

Data was collected anonymously in English, French, Spanish, Portuguese and Arabic languages using close-ended questions. The French, Portuguese Spanish, and Arabic were translated and back translated from the English version. The introduction to the survey included information on the the research team, objectives of the study and the duration required to answer the questionnaire. Following that, there was a section confirming the confidentiality of participants’ responses and highlighting their voluntary participation in the survey. Participants who consented to study participation by ticking a consent form were able to proceed to the survey and answer the questions that followed [32].

### Study variables

#### Covariates

The questionnaire collected information about age at last birthday, sex at birth (male, female), and the country of residence during the COVID-19 pandemic. Country of residence was categorized based on income level according to the World Bank Data Bank into low-income countries (LICs) with a gross national income (GNI) per capita ≤ 1,035 USD in 2019, lower middle-income countries (LMICs) with GNI between 1,036 and 4,045 USD, upper middle-income countries (UMICs) with GNI between 4,046 and 12,535 USD and high-income countries (HICs) with GNI ≥ 12,536 USD [33, 34].

#### Independent variables

Self-care measures used during the pandemic included: talking to friends or family members by phone or through video chat, talking to friends or family face to face, spending time with pets, meditating or doing other mindfulness practices, exercising in or around the house, exercising or spending leisure time outdoors like at a park or walking trail, doing yard work or gardening, participating in creative activities or hobbies (writing, reading, art, crafts), learning a new skill, engaging in distant learning or taking breaks from the news or social media. Respondents were required to check the self-care measures used to cope with the pandemic. The content validity index for this section of the study tool was 0.97 [32].

#### Dependent variables

The two dependent variables for this study were fear of contracting COVID-19 and depression. The measure for fear of contracting COVID-19 was adopted from the Multi-Center AIDS Cohort Study [35]. A question assessed whether the participant experience depression. The question was adapted from the Pandemic Stress Index which measures the psychosocial impact of COVID-19 [36]. Participants selected from eight emotions they may have experienced during COVID-19 by ticking a checkbox against options that were applicable. Only the response for depression was used for this study. The response for experiencing depression was categorized as ‘yes’ if a checkbox was ticked and ‘no’ if no checkbox was ticked. The content validity index for this section of the study tool was 0.90 [32].

### Statistical analysis

Descriptive analyses were conducted for dependent and independent variables. Chi square and t-tests were used to determine the associations between the dependent and independent variables, and the dependent variables and covariates. Two multivariable logistic regression models were built - one for each dependent variable. Adjusted odds ratio (AORs) and 90% confidence intervals (CIs) were calculated. Significance was set at 5%.

## Results

Table 1 shows that of the 2,840 respondents, 1,305 (46.0%) expressed fears of contracting COVID-19 and 599 (21.1%) had indicated they experienced depression. The mean age was 23.4 years (SD = 5.5). The majority of participants were women (71.0%) and from LMICs (52.3%). The most common coping mechanisms used during the pandemic was talking to friends and or family over the phone (60.1%) followed by using video chat (52.8%).

There was a significantly higher proportion of students who reported having fear of contracting COVID-19 than those who did not report this fear who were from LMICs (57.5% vs. 47.8%; p < 0.001), who talked to friends or family over the phone (62.1% and 58.4%, p = 0.044), who participated in creative activities (33.6% and 30.0%, p = 0.040), and who learned new skills or engaged in distant learning (40.3% and 28.3%, p < 0.001).

Also, a significantly higher proportion of respondents reporting experiencing depression than those not reporting experiencing depression were from HICs and UMICs (30.1% vs. 23.2% and 26.2% vs. 20.5%, p < 0.001); talked to friends and or family in person (42.7% and 35.3%, p = 0.001), spent time with pets (23.9% and 15.6%, p < 0.001), did not learn new skills or engage in distant learning (71.5% and 64.7%, p = 0.002), and took breaks from news and social media (33.4% and 27.4%, p = 0.004).

**Table 1:** Factors associated with fear of contracting COVID-19 and experiencing depression among students (N = 2840)

Variables		Total n (%)	Fear of contracting COVID-19			Experiencing depression		
			No: n (%)	Yes: n (%)	p value	No: n (%)	Yes: n (%)	p value
			1535 (54.0)	1305 (46.0)		2241 (78.9)	599 (21.1)	
Age mean (SD)		23.4 (SD 5.5)	23.4 (SD 5.3)	23.3 (SD 5.4)	0.703	23.4 (SD 5.5)	23.2(SD 5.0)	0.492
**Gender**	Woman	2017 (71.0)	1067 (69.5)	950 (72.8)	0.054	1579 (70.5)	438 (73.1)	0.202
	Man	823 (29.0)	468 (30.5)	355 (27.2)		662 (29.5)	161 (26.9)	
**Country income level**	LIC	38 (1.3)	25 (1.6)	13 (1.0)	< 0.001	24 (1.1)	14 (2.3)	< 0.001
	LMIC	1484 (52.3)	733 (47.8)	751 (57.5)		1236 (55.2)	248 (41.4)	
	UMIC	617 (21.7)	369 (24.0)	248 (19.0)		460 (20.5)	157 (26.2)	
	HIC	701 (24.7)	408 (26.6)	293 (22.5)		521 (23.2)	180 (30.1)	
**Talk to friends or family on the phone**	No	1132 (39.9)	638 (41.6)	494 (37.9)	0.044	914 (40.8)	218 (36.4)	0.051
	Yes	1708 (60.1)	897 (58.4)	811 (62.1)		1327 (59.2)	381 (63.6)	
**Talk to friends or family through video chat**	No	1340 (22.3)	740 (48.2)	600 (46.0)	0.235	1057 (47.2)	283 (47.2)	0.973
	Yes	1500 (52.8)	795 (51.8)	705 (54.0)		1184 (52.8)	316 (52.8)	
**Talk to friends or family face to face, in person**	No	1792 (63.1)	963 (62.7)	829 (63.5)	0.664	1449 (64.7)	343 (57.3)	0.001
	Yes	1048 (36.9)	572 (37.3)	476 (36.5)		792 (35.3)	256 (42.7)	
**Spend time with pets**	No	2348 (82.7)	1273 (82.9)	1075 (82.4)	0.696	1892 (84.4)	456 (76.1)	< 0.001
	Yes	492 (17.3)	262 (17.1)	230 (17.6)		349 (15.6)	143 (23.9)	
**Meditate or other mindfulness practices**	No	2349 (82.7)	1289 (84.0)	1060 (81.2)	0.054	1868 (83.4)	481 (80.3)	0.079
	Yes	491 (17.3)	246 (16.0)	245 (18.8)		373 (16.6)	118 (19.7)	
**Exercise in or around your home**	No	1780 (62.7)	954 (62.1)	826 (63.3)	0.529	1414 (63.1)	366 (61.1)	0.370
	Yes	1060 (37.3)	581 (37.9)	479 (36.7)		827 (36.9)	233 (38.9)	
**Exercise or spend leisure time outdoors like at a park or walking trail**	No	2153 (75.8)	1172 (76.4)	981 (75.2)	0.465	1708 (76.2)	445 (74.3)	0.328
	Yes	687 (24.2)	363 (23.6)	324 (24.8)		533 (23.8)	154 (25.7)	
**Do yardwork or gardening**	No	2540 (89.4)	1375 (89.6)	1165 (89.3)	0.792	2010 (89.7)	530 (88.5)	0.392
	Yes	300 (10.6)	160 (10.4)	140 (10.7)		231 (10.3)	69 (11.5)	
**Participate in creative activities or hobbies (writing, reading, art, crafts)**	No	1942 (68.4)	1075 (70.0)	867 (66.4)	0.040	1534 (68.5)	408 (68.1)	0.874
	Yes	898 (31.6)	460 (30.0)	438 (33.6)		707 (31.5)	191 (31.9)	
**Learn a new skill or engage in distant learning**	No	1879 (66.2)	1100 (71.7)	779 (59.7)	< 0.001	1451 (64.7)	428 (71.5)	0.002
	Yes	961 (33.8)	435 (28.3)	526 (40.3)		790 (35.3)	171 (28.5)	
**Taking breaks from the news or social media**	No	2026 (71.3)	1100 (71.7)	926 (71.0)	0.680	1627 (72.6)	399 (66.6)	0.004
	Yes	814 (28.7)	435 (28.3)	379 (29.0)		614 (27.4)	200 (33.4)	

*statistically significant < 0.05

Table 2 shows that those who reported learning a new skill had significantly higher odds of reporting fears of contracting COVID-19 (AOR = 1.669, p < 0.001). In addition, living in LMICs vs. LICs (AOR = 0.330; p = 0.001), talking to friends and or family through video chat (AOR = 0.809; p = 0.049), and learning new skill or engaging in distance learning (AOR = 0.684; p = 0.001) were associated with significantly lower odds of reporting experiencing depression. Those who spent time with pets (AOR = 1.470; p = 0.002), and took breaks from news or social media (AOR = 1.242; p = 0.045) had significantly higher odds of reporting experiencing depression.

**Table 2:** Multivariable logistic regression to determine the associations between fear of contracting COVID-19, and experiencing depression among students (N = 2840)

Variables	Fear of contracting COVID-19				Experiencing depression			
	AOR	95% confidence interval		p value	AOR	95% confidence interval		p value
		Lower	Upper			Lower	Upper	
**Age**	1.006	0.991	1.021	0.423	0.983	0.965	1.001	0.065
**Gender (Ref: Man)**	1.172	0.992	1.386	0.063	1.116	0.907	1.373	0.298
**Country income level (Ref: LIC)**	-	-	-	-	-	-	-	-
**LMIC**	1.886	0.951	3.740	0.069	0.330	0.167	0.653	0.001
**UMIC**	1.199	0.596	2.411	0.611	0.521	0.260	1.045	0.066
**HIC**	1.265	0.630	2.541	0.509	0.567	0.283	1.135	0.109
**Talk to friends or family on the phone (Ref: No)**	1.133	0.956	1.342	0.148	1.188	0.963	1.464	0.108
**Talk to friends or family through video chat (Ref: No)**	1.079	0.909	1.281	0.385	0.809	0.656	0.999	0.049
**Talk to friends or family face to face, in person (Ref: No)**	0.942	0.793	1.119	0.497	1.231	1.000	1.514	0.050
**Spend time with pets (Ref: No)**	1.082	0.875	1.339	0.465	1.470	1.156	1.870	0.002
**Meditate or other mindfulness practices (Ref: No)**	1.117	0.904	1.381	0.306	1.170	0.910	1.505	0.220
**Exercise in or around your home (Ref: No)**	0.902	0.757	1.074	0.247	0.910	0.736	1.125	0.383
**Exercise or spend leisure time outdoors like at a park or walking trail (Ref: No)**	1.033	0.850	1.2–56	0.743	0.976	0.770	1.237	0.842
**Do yardwork or gardening (Ref: No)**	0.971	0.755	1.249	0.821	1.017	0.753	1.373	0.914
**Participate in creative activities or hobbies (Ref: No)**	1.005	0.839	1.203	0.959	0.945	0.758	1.177	0.612
**Learn a new skill or engage in distant learning (Ref: No)**	1.669	1.410	1.975	< 0.001	0.684	0.552	0.848	0.001
**Taking breaks from the news or social media (Ref: No)**	0.974	0.815	1.164	0.771	1.242	1.005	1.536	0.045

*statistically significant < 0.05

## Discussion

The study findings indicate that only one of the 11 self-care measures (learning a new skill or engaging in distant learning) was associated with depression and also associated with fear of contracting COVID-19, though the directions of the associations differed: learning a new skill or engaging in distant learning seems to be associated with lower odds of experiencing depression while it seems to be used by those with a higher likelihood of fear of contracting COVID-19 In addition, students who talked with family or friends through video chat and who lived in LMICs countries seem less likely to report experiencing depression, while students who spent time with pets and took breaks from social media or news seem more likely to report experiencing depression. The study findings partially supported the study hypotheses.

This study provides new information on self-care approaches adopted by students during the COVID-19 pandemic. It also indicates that mental health problems among college students may influence the use of self-care measures. The information can help in the design of appropriate strategies to reduce the risks of college students experiencing depression during crisis like the pandemic. The study findings can also promote new research about the neurobiological pathways to understand how self-care measures affect depression and fear of disease contagion. One of the strengths of this study is the large sample size and the global diversity of the undergraduate and postgraduate students included in the study. The study, however, was cross-sectional in design and cannot support causality or establish time precedence. It also used self-reporting which is subject to response biases such as socially desirable responding, and method variance effects [17]. However, the use of a single self-reporting question to measure depression had been shown to be sensitive, specific and reliable with good agreement with the diagnosis made by psychiatrists [37]. Despite these limitation, there were three findings that may be important for designing and implementing support interventions for undergraduate and postgraduate students during the pandemic.

First, we identified some self-coping mechanisms that were associated with lower odds of reporting experiencing depression. We observed that students who talked to friends or family through video chat, and those who learned a new skill or engage in distant learning had lower odds of reporting experiencing depression. This suggests that students who experienced depression may engage in self-coping mechanisms that enable them to socialize during the pandemic. This findings are not unusual as socializing through video chat and learning new skills help persons with depression rebuild their self-esteem [38, 39].

Students who spent time with pets and who took breaks from the news or social media had higher odds of reporting experiencing depression. It is probable that these students used these coping strategies when they experienced depression. Prior studies indicated a link between increased use of social media and a greater risk of depression [40, 41]. Also people with depression spend less time with social interactions [42]. Pets can provide companionship, boost self-confidence, provide a daily routine for pet owners, promote socialisation and reduce feelings of loneliness [43, 44].

Second, students learning new skills or participating in distance learning had higher odds of reporting fear of COVID-19. The lockdown resulted in many students staying away from school and needing to engage in new modes of learning such as online schooling or other forms of learning that required new skills. The result may reflect an ongoing association of students’ fear about contracting COVID-19 and the changes resulting in their lifestyle because of the pandemic rather than an active engagement with skills building and creative activities to moderate the feeling of fear during the pandemic. Otherwise learning new skills and engaging in creative activities are forms of distractions that interfere with the mechanisms required for fear by preventing emotional and/or cognitive processing of the fear stimulus and response [45]. In the long-term, distraction may have a relieving effect on fear of COVID-19 because of the potential of causing the individual to relax while engaged with the task [45]. The usefulness of learning new skills or participating in distance learning as distractive approaches is the collarity effect of reducing the depression as observed in this study. Further long-term studies are needed to explore our hypothesis.

Finally, when compared to LICs, students from LMICs, UMICs and HICs had higher odds of reporting depression though this difference was significant only for LMICs. This has significant implications for the mental health of students as the findings imply that country-specific context may be associated with higher risk of mental health problems. The COVID-19 pandemic may have had worse impact on the education system of those in LICs. Prior studies have indicated that dramatic changes in physical activity, sleep, and time use at the onset of the COVID-19 pandemic as a major cause of depression among students [32, 46]. The present study suggests that access to mental health supportive systems may be less accessible in LICs than other countries, especially LMICs. There is scarce data about differences in mental health problems for students among countries. Such studies may help explain the reasons for the differences observed in this study.

The study findings suggest that during pandemics like that of the COVID-19, opportunities for students to actively hold audio or video conversations with their family/friends should be promoted. Students should also be encouraged to learn new skills or engage in distance learning. Attention should be paid to the care of students in LICs, those who spend time with pets and those who take breaks from news or social media as these may be warning signs of experiencing depression.

## Conclusions

Some self-care strategies may be effective in ameliorating the impact of the COVID-19 pandemic on the mental health of students. Self-care strategies that are distractive and long-term like learning new skills place cognitive demands on students thereby they are significantly associated with lower risk of reporting experiencing depression. These cognitively demanding strategies were, however, associated with fear of COVID-19. Short-term self-care strategies like talking to family or friends through video chating may also help manage reported depression. Spending time with pets was associated with higher odds of reporting experiencing depression, a possible reflection of someone experiencing depression who is trying to relieve loneliness. Special attention needs to be paid to students in LICs to support their mental health and wellness during the COVID-19 pandemic.

## Data Availability

All data are available from the corresponding author upon reasonable request.

## Abbreviations

LICs: Low-income countries
GNI: Gross national
LMICs: Lower middle-income countries
UMICs: Upper middle-income countries
HICs: High-income countries
AOR: Adjusted odds ratio
CI: Confidence intervals

## References

[1] Di Domenico L, Pullano G, Sabbatini CE, Boëlle PY, Colizza V (2020). Impact of lockdown on COVID-19 epidemic in Île-de-France and possible exit strategies. BMC Med.

[2] Choi B, Jegatheeswaran L, Minocha A, Alhilani M, Nakhoul M, Mutengesa E (2020). The impact of the COVID-19 pandemic on final year medical students in the United Kingdom: a national survey. BMC Med Educ.

[3] Ahmed S, Mvalo T, Akech S, Agweyu A, Baker K, Bar-Zeev N (2020). Protecting children in low-income and middle-income countries from COVID-19. BMJ Glob Health.

[4] Li HY, Cao H, Leung DYP, Mak YW (2020). The psychological impacts of a COVID-19 outbreak on college students in China: a longitudinal study. Int J Environ Res Public Health.

[5] Coetzee BJ, Kagee A (2020). Structural barriers to adhering to health behaviours in the context of the COVID-19 crisis: considerations for low- and middle-income countries. Glob Public Health.

[6] Zhai Y, Du X (2020). Addressing collegiate mental health amid COVID-19 pandemic. Psychiatry Res.

[7] Skapinakis P, Bellos S, Oikonomou A, Dimitriadis G, Gkikas P, Perdikari E, Mavreas V (2020). Depression and its relationship with coping strategies and illness perceptions during the COVID-19 lockdown in Greece: a cross-sectional survey of the population. Depress Res Treat.

[8] Sandhu P, de Wolf M (2020). The impact of COVID-19 on the undergraduate medical curriculum. Med Educ Online.

[9] Kochuvilayil T, Fernandez RS, Moxham LJ, Lord H, Alomari A, Hunt L (2021). COVID-19: knowledge, anxiety, academic concerns and preventative behaviours among australian and indian undergraduate nursing students: a cross-sectional study. J Clin Nurs.

[10] Singh S, Roy D, Sinha K, Parveen S, Sharma G, Joshi G (2020). Impact of COVID-19 and lockdown on mental health of children and adolescents: a narrative review with recommendations. Psychiatry Res.

[11] Rehman U, Shahnawaz MG, Khan NH, Kharshiing KD, Khursheed M, Gupta K (2021). Depression, anxiety and stress among Indians in times of Covid-19 lockdown. Community Ment Health J.

[12] Burns D, Dagnall N, Holt M (2020). Assessing the impact of the COVID-19 pandemic on student wellbeing at universities in the United Kingdom: a conceptual analysis. Front Educ.

[13] Zhai Y, Du X (2020). Mental health care for international chinese students affected by the COVID-19 outbreak. Lancet Psychiatry.

[14] Son C, Hegde S, Smith A, Wang X, Sasangohar F (2020). Effects of COVID-19 on college students’ mental health in the United States: interview survey study. J Med Internet Res.

[15] Richardson T, Elliott P, Roberts R, Jansen M (2017). A longitudinal study of financial difficulties and mental health in a national sample of british undergraduate students. Community Ment Health J.

[16] Schlesselman LS, Cain J, DiVall M (2020). Improving and restoring the well-being and resilience of pharmacy students during a pandemic. Am J Pharm Educ.

[17] Rodríguez-Hidalgo AJ, Pantaleón Y, Dios I, Falla D (2020). Fear of COVID-19, stress, and anxiety in university undergraduate students: a predictive model for depression. Front Psychol.

[18] Lyons Z, Wilcox H, Leung L, Dearsley O (2020). COVID-19 and the mental well-being of australian medical students: impact, concerns and coping strategies used. Australas Psychiatry.

[19] Lai AY, Lee L, Wang MP, Feng Y, Lai TT, Ho LM (2020). Mental health impacts of the COVID-19 pandemic on international university students, related stressors, and coping strategies. Front Psychiatry.

[20] Abdulghani HM, Sattar K, Ahmad T, Akram A (2020). Association of COVID-19 pandemic with undergraduate medical students’ perceived stress and coping. Psychol Res Behav Manag.

[21] Birken SA, Haines ER, Hwang S, Chambers DA, Bunger AC, Nilsen P (2020). Advancing understanding and identifying strategies for sustaining evidence-based practices: a review of reviews. Implement Sci.

[22] Terroni L, Fraguas R (2010). Depression affecting moral judgment. Behav Brain Sci.

[23] Husain N, Gater R, Tomenson B, Creed F (2004). Social factors associated with chronic depression among a population-based sample of women in rural Pakistan. Soc Psychiatry Psychiatr Epidemiol.

[24] Beattie G. Social Causes of Depression. Rochester Institute of Technology. 2005. Available at: http://www.personalityresearch.org/papers/beattie.html. Updated November, 2005. Accessed December 20, 2021.

[25] Vikram Patel (2001). Cultural factors and international epidemiology: Depression and public health. Br Med Bull.

[26] Folayan MO, Ibigbami O, Brown B, El Tantawi M, Uzochukwu B, Ezechi OC (2022). Differences in COVID-19 preventive behavior and food insecurity by HIV statusin Nigeria. AIDS Behav.

[27] Statistica. Percentage of world population with select mental health disorders as of 2019. https://www.statista.com/statistics/979852/prevalence-of-mental-health-disorders-globally/.

[28] Eng J (2003). Sample size estimation: how many individuals should be studied?. Radiology.

[29] Mirzaei A, Carter SR, Patanwala AE, Schneider CR (2022). Missing data in surveys: key concepts, approaches, and applications. Res Social Administrative Pharm.

[30] Wilson VanVoorhis CR, Morgan BL (2007). Understanding power rules of thumb for determining sample sizes. Tutorials in Quantitative Methods for Psychology.

[31] Boni Raquel Brandini De (2020). Web surveys in the time of COVID-19. Cad. Saúde Pública.

[32] Ellakany P, Zuñiga RAA, El Tantawi M, Brown B, Aly NM, Ezechi OC (2022). Impact of the COVID-19 pandemic on student’ sleep patterns, sexual activity, screen use, and food intake: a global survey. PLoS ONE.

[33] World Bank. World Bank Country and Lending Groups. 2020. Available at: https://datahelpdesk.worldbank.org/knowledgebase/articles/906519-world-bank-country-and-lending-groups. Accessed Decemeber 20, 2021.

[34] Raghupathi V, Raghupathi W (2020). Healthcare expenditure and economic performance: insights from the United States data. Front Public Health.

[35] Kaslow RA, Ostrow DG, Detels R, Phair JP, Polk BF, Rinaldo CR (1987). The multicenter AIDS cohort study: rationale, organization, and selected characteristics of the participants. Am J Epidemiol.

[36] Harkness A. The pandemic stress index. University of Miami; 2020.

[37] Santos BF, Oliveira HN, Miranda AES, Hermsdorff HHM, Bressan J, Vieira JCM (2021). Research quality assessment: reliability and validation of the self-reported diagnosis of depression for participants of the cohort of universities of Minas Gerais (CUME project). J Affect Disorders Rep.

[38] Elmer T, Stadtfeld C (2020). Depressive symptoms are associated with social isolation in face-to-face interaction networks. Sci Rep.

[39] Bulut S (2019). Socialization helps the treatment of depression in modern life. Open J Depress.

[40] Gao J, Zheng P, Jia Y, Chen H, Mao Y, Chen S (2020). Mental health problems and social media exposure during COVID-19 outbreak. PLoS ONE.

[41] Zhao N, Zhou G (2020). Social media use and mental health during the COVID-19 pandemic: moderator role of disaster stressor and mediator role of negative affect. Appl Psychol Health Well Being.

[42] Su Z, McDonnell D, Wen J, Kozak M, Abbas J, Šegalo S (2021). Mental health consequences of COVID-19 media coverage: the need for effective crisis communication practices. Global Health.

[43] Mental Health Foundation. Pets and mental health. 2021 Available at: https://www.mentalhealth.org.uk/a-to-z/p/pets-and-mental-health. Accessed Decemeber 20, 2021. Updated May 18, 2021. Accessed December 20, 2021.

[44] Blurt team. Depression: why spending time with animals might help. Increasing awareness and understanding of depression. 2021. Available at: https://www.blurtitout.org/2017/05/12/depression-why-animals-help/. Accessed December 29, 2021.

[45] Rodriguez BI, Craske MG (1993). The effects of distraction during exposure to phobic stimuli. Behav Res Ther.

[46] Shah SMA, Mohammad D, Qureshi MFH, Abbas MZ, Aleem S (2021). Prevalence, psychological responses and associated correlates of depression, anxiety and stress in a global population, during the coronavirus disease (COVID-19) pandemic. Community Ment Health J.

